# Study of the shared gene signatures of polyarticular juvenile idiopathic arthritis and autoimmune uveitis

**DOI:** 10.3389/fimmu.2023.1048598

**Published:** 2023-03-08

**Authors:** Jie Zheng, Yong Wang, Jun Hu

**Affiliations:** Department of Pediatrics, Fujian Medical University Union Hospital, Fuzhou, Fujian, China

**Keywords:** arthritis, juvenile rheumatoid, autoimmune uveitis, genetic effects, immune system disorders, neutrophil degranulation

## Abstract

**Objective:**

To explore the shared gene signatures and potential molecular mechanisms of polyarticular juvenile idiopathic arthritis (pJIA) and autoimmune uveitis (AU).

**Method:**

The microarray data of pJIA and AU from the Gene Expression Omnibus (GEO) database were downloaded and analyzed. The GEO2R tool was used to identify the shared differentially expressed genes (DEGs) and genes of extracellular proteins were identified among them. Then, weighted gene co-expression network analysis (WGCNA) was used to identify the shared immune-related genes (IRGs) related to pJIA and AU. Moreover, the shared transcription factors (TFs) and microRNAs (miRNAs) in pJIA and AU were acquired by comparing data from HumanTFDB, hTFtarget, GTRD, HMDD, and miRTarBase. Finally, Metascape and g: Profiler were used to carry out function enrichment analyses of previously identified gene sets.

**Results:**

We found 40 up-regulated and 15 down-regulated shared DEGs *via* GEO2R. Then 24 shared IRGs in positivity-related modules, and 18 shared IRGs in negatively-related modules were found after WGCNA. After that, 3 shared TFs (ARID1A, SMARCC2, SON) were screened. And the constructed TFs-shared DEGs network indicates a central role of ARID1A. Furthermore, hsa-miR-146 was found important in both diseases. The gene sets enrichment analyses suggested up-regulated shared DEGs, TFs targeted shared DEGs, and IRGs positivity-correlated with both diseases mainly enriched in neutrophil degranulation process, IL-4, IL-13, and cytokine signaling pathways. The IRGs negatively correlated with pJIA and AU mainly influence functions of the natural killer cell, cytotoxicity, and glomerular mesangial cell proliferation. The down-regulated shared DEGs and TFs targeted shared DEGs did not show particular functional enrichment.

**Conclusion:**

Our study fully demonstrated the flexibility and complexity of the immune system disorders involved in pJIA and AU. Neutrophil degranulation may be considered the shared pathogenic mechanism, and the roles of ARID1A and MiR-146a are worthy of further in-depth study. Other than that, the importance of periodic inspection of kidney function is also noteworthy.

## 1 Introduction

Juvenile idiopathic arthritis (JIA) is the most common chronic rheumatic disease in children, including a group of diseases with different genetic backgrounds, etiology, and outcomes. Due to a large heterogeneity and unknown etiology, its classification criteria have been modified several times with the changing of people’s recognition, and the latest edition is still in progress (1). Autoimmune uveitis (AU) is a complex intraocular inflammatory disease caused by several etiological entities. Some of the uveitis’ entities manifest nonspecific or atypical clinical presentation. The majority of pediatric uveitis is noninfectious uveitis. JIA is the most commonly associated condition with noninfectious uveitis. Likewise, chronic anterior uveitis that affects predominantly the iris and ciliary body is a common extra-articular manifestation of JIA (2).

Polyarticular juvenile idiopathic arthritis(pJIA) is the second most common form of JIA, with chronic anterior uveitis as its most common and severe extra-articular manifestation (3, 4). Owning to insidious symptoms of eye damage, it is strongly suggested that slit lamp examination should be performed frequently for quite a long time (every 3 months for at least 5 years) in early onset JIA patients to diagnose uveitis before complications develop (5). Hence, it is of clinical and economic significance to identify uveitis early if comorbid with JIA.

With advances in sequencing and genotyping methodology, genetic studies have enhanced our understanding of disease etiology. The interplay of genetic and environmental factors is considered important for the onset of both diseases. For example, they have been reported to both be strongly associated with major histocompatibility complex (MHC) genes that encode the α chains of the MHC class I molecules human leukocyte antigen (HLA)-A, HLA-B, and HLA-C and the α and β chains of the MHC class II molecules HLA-DR, HLA-DP, HLA-DQ. Studies from several GWAS reveal some shared alleles between uveitis and JIA, especially in HLA-DR loci such as HLA-DRB1*11, *13 and HLA-DR9, HLA-B27 (6–8).

Other than MHC genes, genetic factors affecting IL-23/Th17 pathway, Th2/Th1 cytokine balance, PD-1/PD-L1, NF-κB signaling pathway, and NOD2 signaling pathway, etc. have also been reported in both diseases (2, 9, 10). Notably, growing pieces of evidence have supported the significant role of the IL-23/IL-17/G-CSF axis. The most common susceptibility gene of several major noninfectious uveitis outside the MHC region is IL23R, identified from some recent GWASs studies (11). Moreover, peripheral blood mononuclear cells (PBMCs) IL-23 protein and mRNA levels, serum IL-17 level, IL-17A+ cells in the lamina propria were found to be elevated in several different types of active noninfectious uveitis patients, and IL-23/Th17 pathway cells are thought to be the basis of a common pathophysiology of noninfectious uveitis. Likewise, IL17+ T cells were found to be enriched within the joints and peripheral blood of patients with active JIA (12). It has been found in JIA patients that naive CD4+ cells can inappropriately become IL-17 producers under polarizing conditions (13). The IL-23 receptor is not only a key checkpoint for naive T cell to differentiate into pathogenic Th17 cells, but also contribute to Th17 cell cytokine production, such as IL-17, IL-6, interferon γ, etc (6). Additionally, levels of IL-23 were found upregulated in JIA patients and make a promising biomarker (14).

IL-17 could induce neutrophil-mediated inflammation in animal models of uveitis, and growing bodies of evidence point toward the role of neutrophils in uveitis and JIA (15–18). It has been found that cathelicidin, released from neutrophil degranulation, could not only induce Th17 cell differentiation but also promotes the survival of Th17 cells (19). On the other hand, neutrophils also exhibit autocrine IL-17 activity following IL-6 and IL-23 stimulation (20). Considering more effects of neutrophils have been found in the immune regulation process, its role may mature the theory of immune disorders in both diseases.

Due to the rapid progress of gene microarray technology, the expression of a great many gene data can be analyzed conveniently. Thus, we tried to use the weighted gene co-expression network analysis (WGCNA), which has been successfully applied in various biological contexts to identify common risk genes within multiple disease phenotypes, to explore shared gene signatures associated with pJIA and uveitis in wish to gain a better understanding of their pathogenesis including the participating of MHC, IL, and neutrophils from the genetic level.

## 2 Materials and methods

### 2.1 GEO datasets download and processes

We used the Medical Subject Headings “Arthritis, Juvenile Rheumatoid polyarticular onset” or “autoimmune uveitis” to search JIA and uveitis gene expression profiles in the GEO database. PBMCs form a critical part of the immune system which is important in the pathogenesis of pJIA and AU. Hence, we searched gene expression files that used PBMC as their sequencing organization to study their underline mechanism. The obtained datasets were filtered by the following criteria: 1. the gene expression profiling must include pJIA, AU, and controls. 2. the organization used for sequencing should be PBMC. 3. the number of samples used for WGCNA should be more than 15 to ensure the accuracy of the WGCNA. Finally, the GEO datasets numbered GSE66936 (21) and GSE55319 (22) were selected. **Table 1** provides a detailed overview of their information.

**Table 1:** Information of GEO datasets selected.

GSE number	Platform	Samples	Source	Disease
GSE66936	GPL570	4 patients VS 17 controls	PBMC	AU
GSE55319	GPL6884	26 patients VS 19 controls	PBMC	pJIA

### 2.2 Identification of shared differentially expressed genes

GEO2R (https://www.ncbi.nlm.nih.gov/geo/geo2r), the official tool of the GEO database based on linear models for microarray analysis for comparing samples in GEO to identify differentially expressed genes (DEGs) across experimental conditions, was used to explore the DEGs in GSE66936 and GSE55319 to find the shared genetic effects of pJIA and AU. To provide a good balance between the discovery of statistically significant genes and the limitation of false positives, the Benjamini-Hochberg false discovery rate method is selected and an adjusted “P value < 0.05” was set as the threshold for screening of the DEGs.

### 2.3 Weighted gene co-expression network analysis of immune-related genes

ImmPort was a database that collects data from clinical and mechanistic studies with a primary focus on allergy, autoimmune diseases, infection responses, transplantation, and vaccine responses created by the National Institute of Allergy and Infectious Diseases (23). The immune-related genes (IRGs) were downloaded from the ImmPort database considering abnormal autoimmunity is both an important feature of pJIA and AU and forms a critical part of their common mechanism. After eliminating duplicate genes, 1793 IRGs were obtained (**Supplementary Table 1**).

Then, we selected the IRGs of GSE66936 and GSE55319 for WGCNA, an algorithm widely used to find co-expression gene modules with high biological significance, to explore the relationship between screened gene networks and diseases according to the spearman correlation coefficient (24). ImageGP (http://www.ehbio.com/ImageGP, accessed around July 2022), a web application with a high-level web framework used for backend data preprocessing and analysis mostly based on the R programming language, was employed to perform it (25). We adjusted the parameters appropriately as follows to avoid redundant modules: the minModuleSize was 25 in the WGCNA of pJIA and 50 in AU, and the deepSplit was 1.5. Other parameters in this study were described below: networkType = “signed” and R square cut = 0.85. Finally, the expression profiles of each module were summarized by the module eigengene and the correlation between the module eigengene and clinical features was calculated. The modules that have a high correlation coefficient with clinical features were focused and the genes in these modules were selected for subsequent analyses.

### 2.4 The common transcription factors- shared DEGs network construction

In order to explore key transcription factors (TFs) from shared DEGs that can affect interested genes expression, a list of 1665 TFs was downloaded from HumanTFDB which is one of the most comprehensive TF databases of genome-wide TFs and cofactors (26) (**Supplementary Table 2**). After that, hTFtarget, a database integrates human TF target resources and related epigenetic modification information from GEO, NCBI Sequence Read Archive, and ENCODE (27), and The Gene Transcription Regulation Database(GTRD) which provides uniform annotation and integrative analyses of all NGS data from GEO and NCBI Sequence Read Archive (28) were used to obtain the targeted genes of interested TFs. The TFs-shared DEGs network was generated by Cytoscape (3.9.0). In the visual network, nodes of ellipse shape represent TFs (who can also be targets), and nodes of diamond shape imply purely targets. Besides, the red color suggests up-regulated genes and the blue color means down-regulated genes.

### 2.5 The common microRNAs-shared DEGs network construction

MicroRNAs (miRNAs) are a group of small non-coding RNAs and have been demonstrated to negatively modulate gene expression. We further explored the roles of miRNAs in the regulation of risk genes in AU and pJIA. HMDD is a database containing experiment-supported evidence for human miRNA and disease associations, in which the terms “Juvenile Rheumatoid Arthritis” and “Uveitis” were selected to obtain the AU-associated and pJIA-associated miRNAs (29). After that, the target genes of hsa-miR-146 were identified by miRTarBase, which is an informative resource for experimentally validated miRNA-target interactions (30).

### 2.6 The function enrichment analysis of gene sets

We used metascape (http://metascape.org) and g: Profiler (https://biit.cs.ut.ee/gprofiler), which are both reliable services based on up-to-date high-quality data for functional annotation, to expound promising signaling pathways correlated with gene sets (31, 32). The specie was limited to “Homo sapiens”, and the cut-off p-value was set as 0.01 for enrichment analysis when doing the Reactome (REAC) enrichment analysis in metascape. The parameters of g: Profiler were set as follows: the specific organism was chosen “H. sapiens”, the tailor-made algorithm “g: SCS threshold” was selected as the method for multiple testing correction, and the user threshold was 0.05. The biological pathways used were the Kyoto Encyclopedia of Genes and Genomes (KEGG), Human Phenotype Ontology (HP), and WikiPathways (WP) databases. The Comprehensive Resource of Mammalian protein complexes (CORUM) was selected as the protein database used for analysis.

## 3 Results

### 3.1 The shared DEGs in pJIA and AU

**Figure 1:**
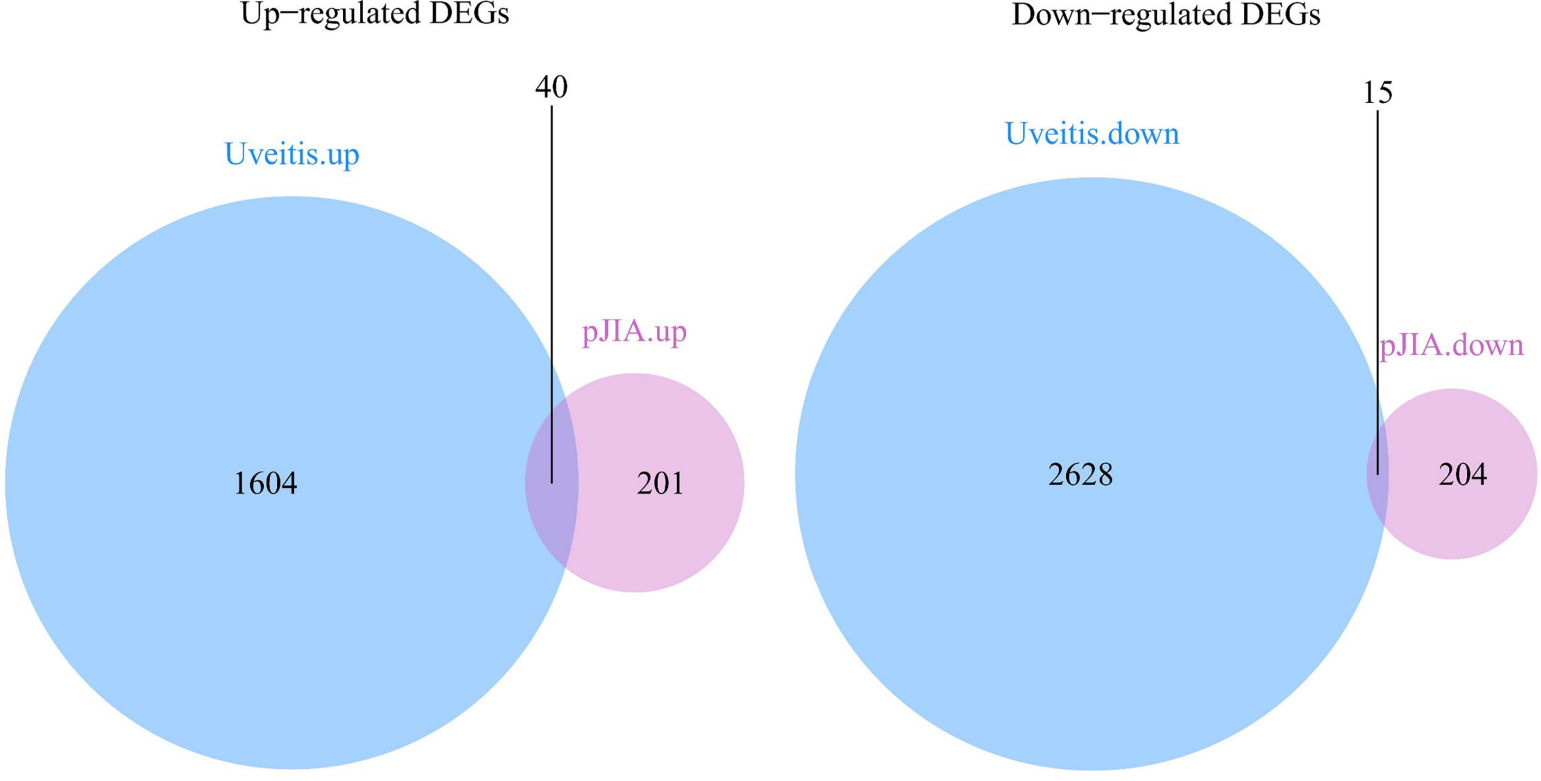
Differentially expressed genes in pJIA and uveitis. Up-regulated shared DEGs: MMP9, IGF2R, CYP4F3, SORL1, LRP10, PLCG2, WNK1, TPP1, ALOX5, FNBP1, ATP6V0A1, WAS, ARID1A, TGOLN2, UBA1, LBR, CAPN1, SYK, IL6R, RBM10, MED16, GAK, ACTN4, SNX27, COPA, CD99L2, APAF1, CBL, GLG1, DEF6, ANKRD11, PRKCSH, SMARCC2, KIDINS220, N4BP1, CNOT1, STK38, FDFT1, WDR37 SON. Down-regulated shared DEGs: RPAIN, DYNLRB1, MRPS18C, TMEM134, SEC61B, CCT2, FAM76B, MGST3, HNRNPC, RABEP1, BOLA2, TTC38, CREBRF, NDUFB7, SUZ12.

**Figure 2:**
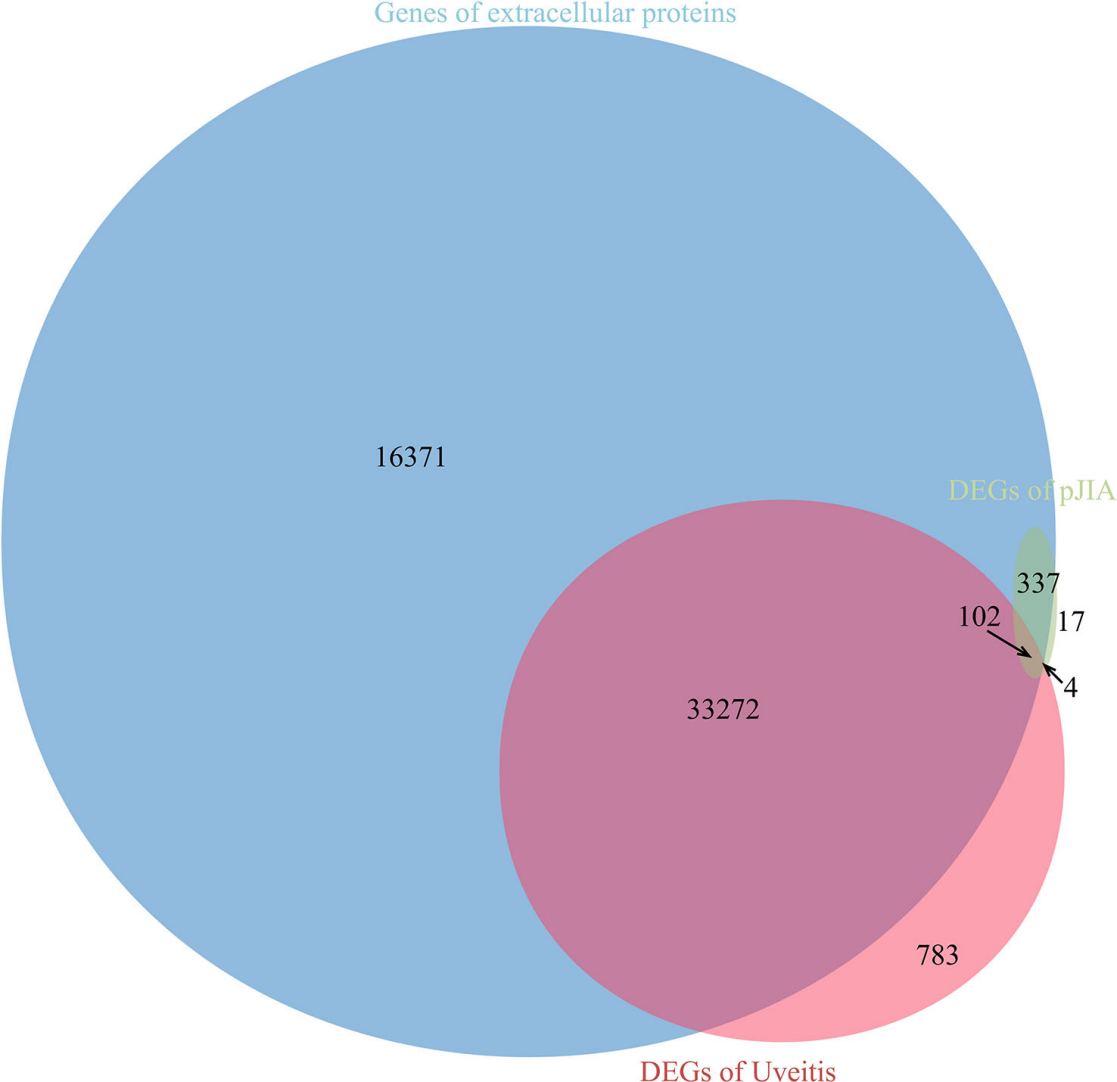
Relationship between differentially expressed genes and genes encoding extracellular proteins.

There were 40 common up-regulated genes and 15 common down-regulated genes in pJIA and AU (**Figure 1**), which were defined as shared DEGs. Then those that code extracellular proteins were scanned. As is shown in **Figure 2**, most DEGs exert their functions extracellularly. Other than that, we found all shared DEGs code extracellular proteins.

### 3.2 The WGCNA of IRGs

**Figure 3:**
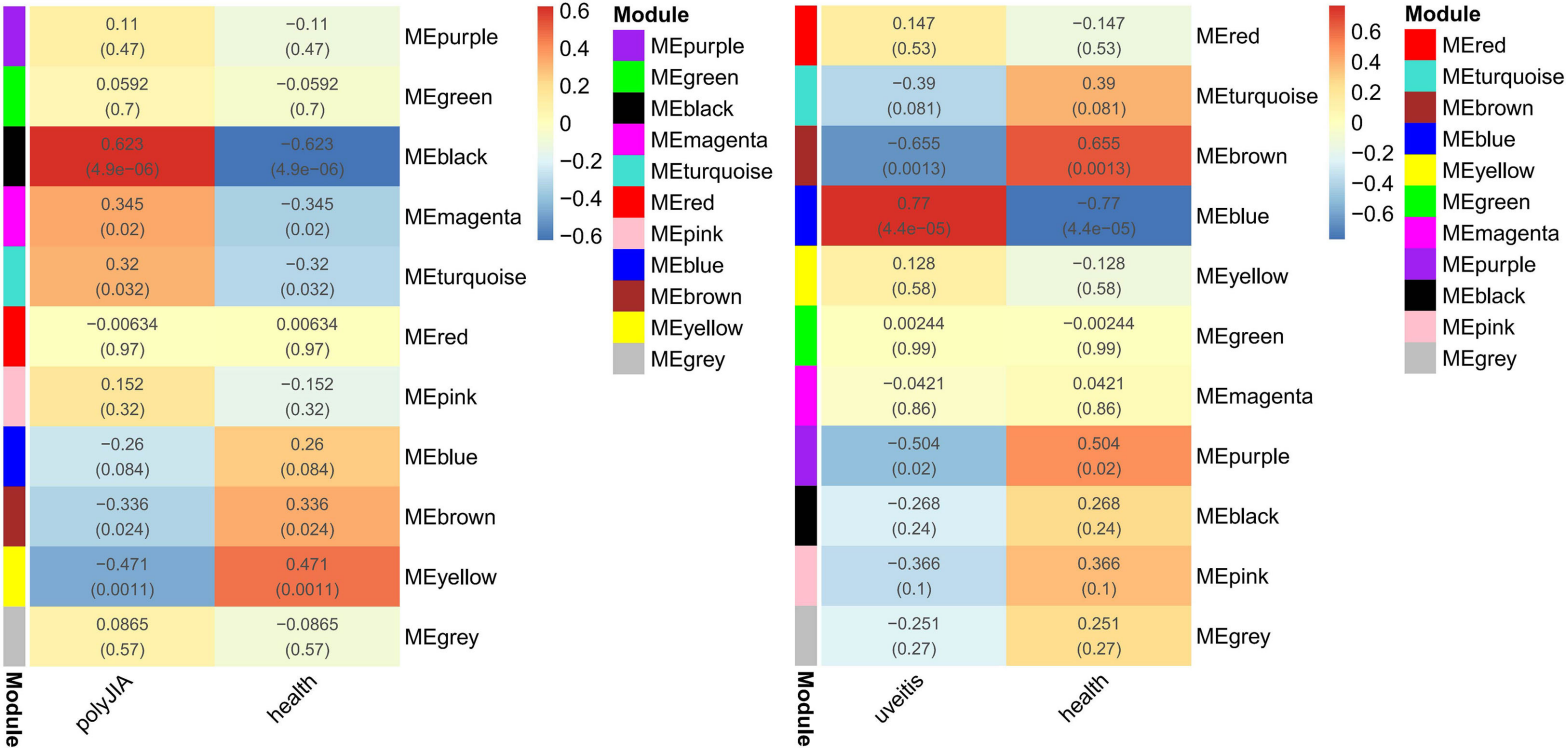
Weighted gene co-expression network analyses concerning immune-related genes associated with pJIA and uveitis. r value (P value); ME, module eigengene.

As shown in **Figure 3**, 11 modules were identified both in AU and pJIA. The heat maps about module–trait relationships that evaluate the associations between each module and disease were displayed. The blue, brown, and purple modules in AU and the yellow and black modules in pJIA were selected for further study respectively according to their spearman’s rank correlation r is more than 0.4(P<0.05).

**Figure 4:**
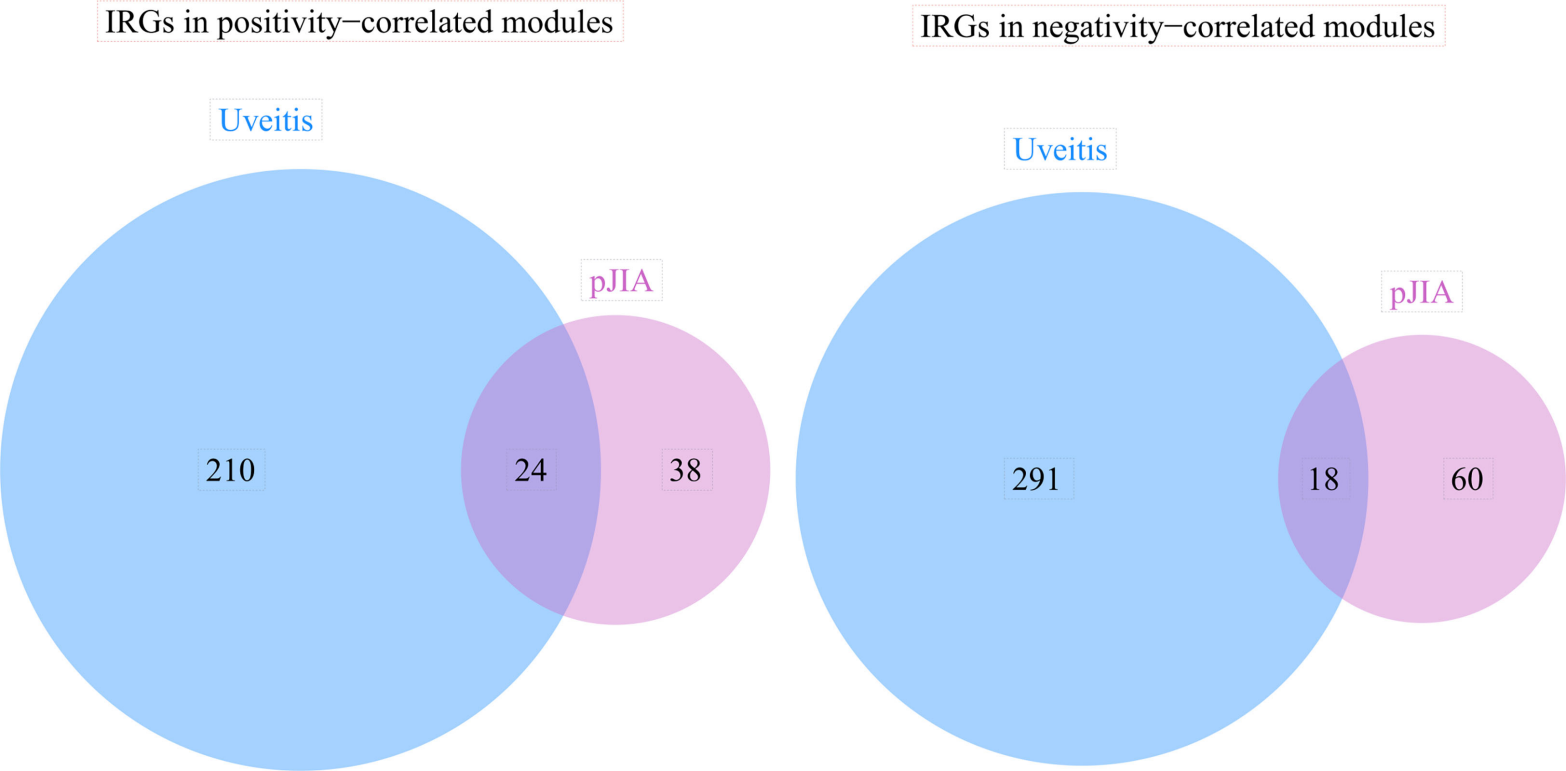
The immune-related genes found correlated with pJIA and uveitis. Shared IRGs in positivity-correlated modules: IGF2R, GRN, ZYX, PLCG2, CYBB, IL4R, TYK2, CD4, IKBKG, ADAR, SYK, TPM2, CMTM7, AHNAK, SEMA4D, INPP5D, ZC3HAV1, CMTM3, VIPR1, PSME3, PSMD2, ACVR1B, ACO1, TGFB1. Shared IRGs in negatively-correlated modules: KIR2DL1, KIR2DL4, NCR3, NENF, KIR3DL1, CXCR6, KIR2DL5A, CACYBP, KIR3DL3, IL22, PDGFD, NCR1, PAEP, GHRHR, RORA, PDGFB, JAG2, IL17C.

The blue module was positively correlated with AU, including 234 genes. And, the brown and purple modules were negatively correlated with AU, including 309 genes in total. Similarly, 62 genes were positively correlated with pJIA in the black module, while 78 genes were negatively correlated with pJIA in the yellow module (**Supplementary Table 3**). There were 24 shared IRGs in positivity-related modules of both diseases, and 18 shared IRGs in negatively-related modules were found (**Figure 4**). Furthermore, by comparing shared DEGs, 6 IRGs (MMP9, IGF2R, PLCG2, SYK, IL6R, CBL) were identified in the up-regulated shared DEGs, while only 1 IRG(RABEP1) was found in the down-regulated shared DEGs.

### 3.3 The common TFs- shared DEGs network construction

**Figure 5:**
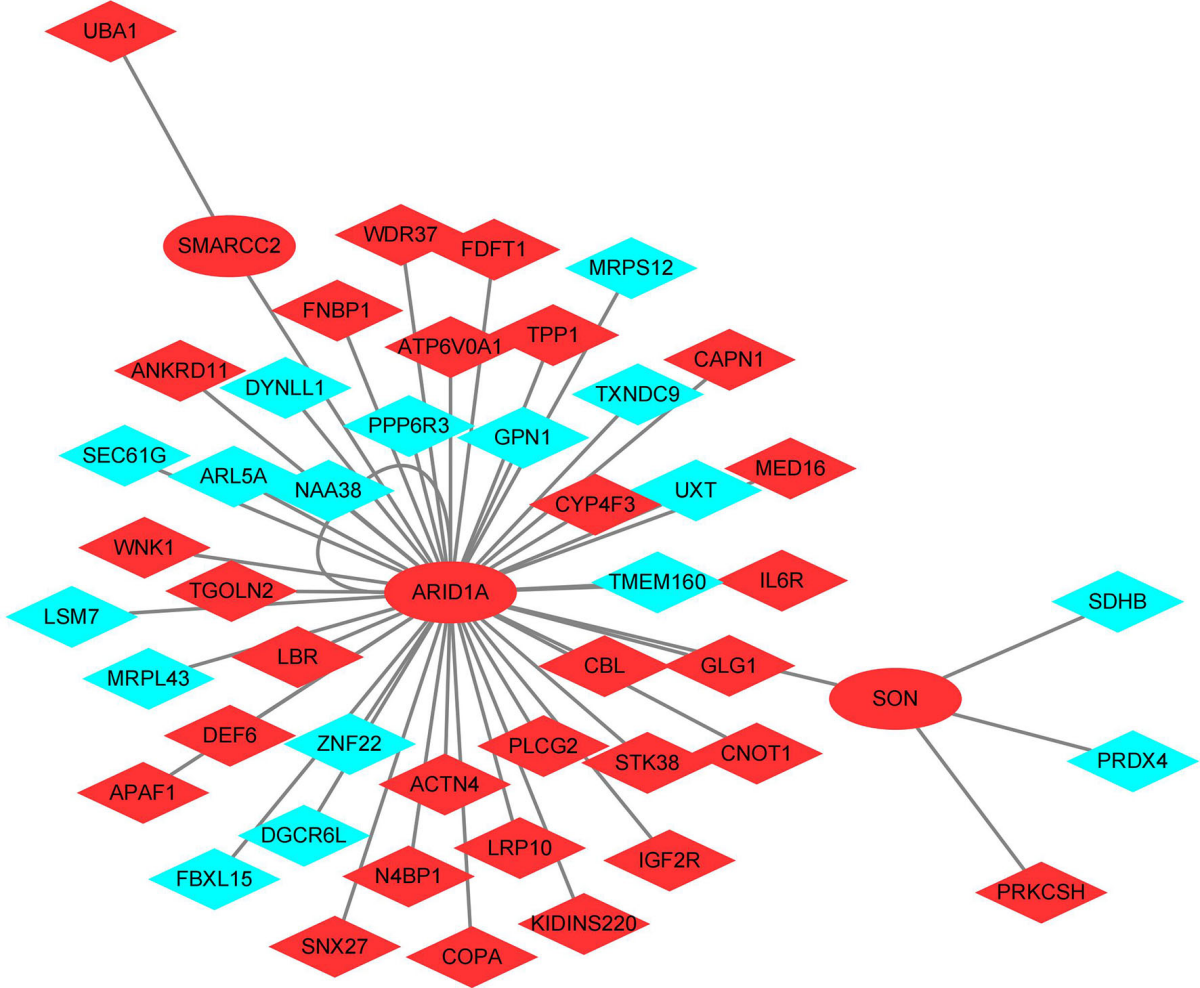
Transcription factors - shared differentially expressed genes regulatory network. Red: Up-regulated shared DEGS; Blue: Down-regulated shared DEGs.

As can be seen from **Figure 5**, ARID1A, SMARCC2, and SON were the TFs identified in the shared DEGs, and they are related to the expression of 45 shared DEGs. There were 32 up-regulated shared DEGS, including the 3 TFs, and 13 down-regulated shared DEGs in the network. ARID1A is at the center of the TFs-shared DEGs network and targets all other TFs.

### 3.4 The miRNA targeted shared DEGs

According to the HMDD database, hsa-miR-146 is the only miRNA that is related to AU and pJIA simultaneously. Among those targeted genes identified by miRTarBase, 131 were up-regulated DEGs of AU, and 20 were that of pJIA. Meanwhile, 120 targeted genes of hsa-miR-146 were down-regulated DEGs of AU, and 16 were that of pJIA (**Supplementary Table 4**).

### 3.5 The functional analyses of gene sets

**Figure 6:**
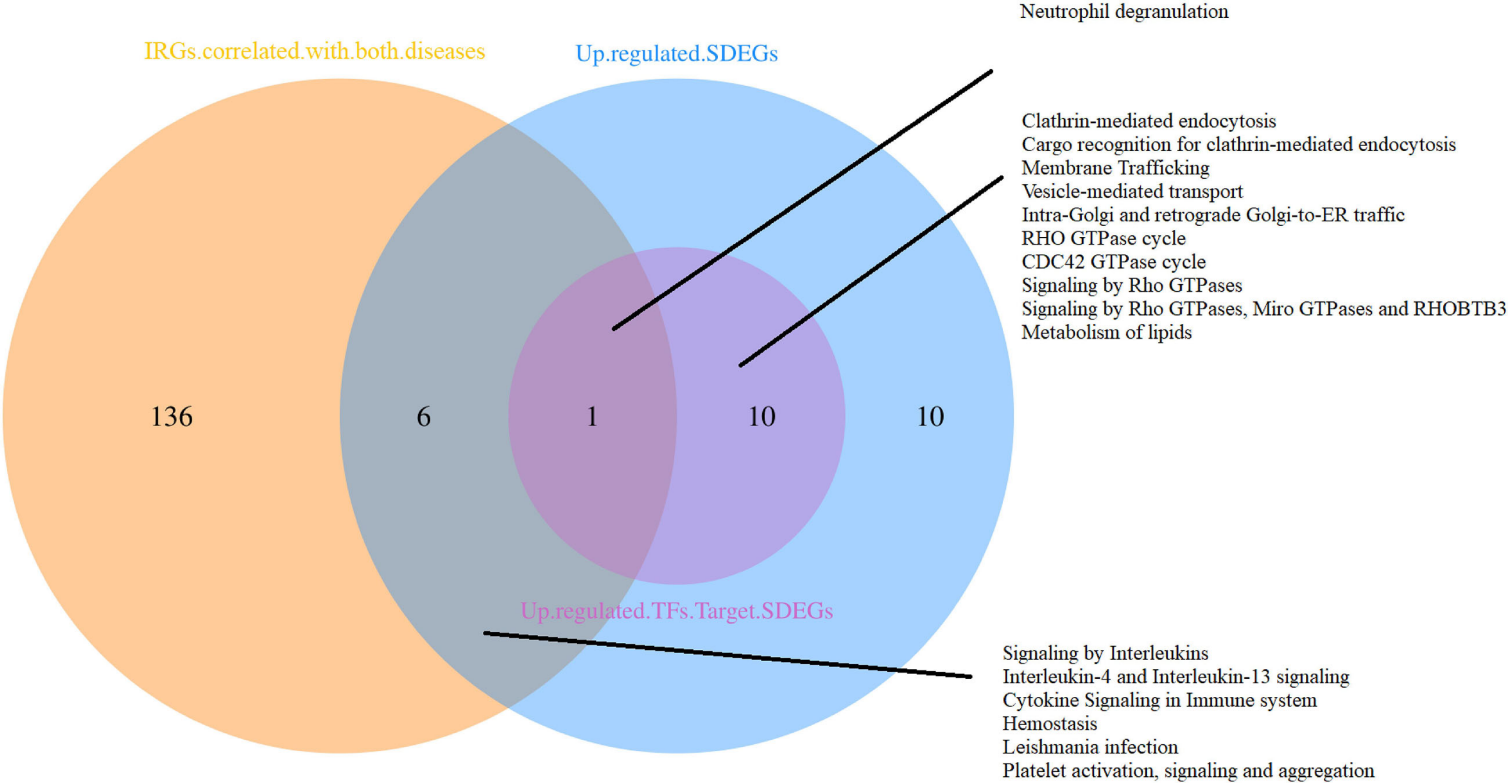
Functional analyses of gene sets positively correlated with both pJIA and uveitis.

The functional analyses of sets positively correlated with both diseases were performed respectively. Results indicate that up-regulated shared DEGs are involved in the functions of IL-4, IL-13, RHO GTPase, Miro GTPases and RHOBTB3, RAC1 GTPase, neutrophil degranulation, and Fc γ receptor(FcγR). The IRGs were mostly enriched related to macrophage activation, expression of chemokine receptors during T-cell polarization, positive regulation of calcium-mediated signaling, IL-3, IL-4, and IL-13 pathway, Th17, Th1, and Th2 cell differentiation, NF-κB, RUNX3, BCR, TCR, FcϵRI, class I MHC, MAPK family signaling cascades, neutrophil degranulation, cell-cell adhesion, etc. Targets of the 3 TFs in the shared DEGs also have a close relationship with RHO GTPase, Miro GTPases, and RHOBTB3, neutrophil degranulation. As shown in **Figure 6**, the neutrophil degranulation process is associated with all the gene sets positively correlated with both diseases.

On the other hand, gene sets negatively correlated with both diseases were also taken into functional analyses, but down-regulated shared DEGs and those TFs targeted genes did not show any particular functional enrichment. However, results imply that the IRGs might influence the functions of natural killer cells and cytotoxicity. Interestingly, they were also enriched in the pathway of glomerular mesangial cell proliferation.

Then we explored the specific function pattern of hsa-miR-146 targeted genes. The up-regulated targeted DEGs in pJIA are related to neutrophil degranulation, Fc, tyrosine kinases, and interleukins, and those in AU were enriched mainly in interferon α/β, interferon γ, Rho GTPases, Miro GTPases, and RHOBTB3, metalloprotease class of deubiquitinases, RUNX1, etc. However, the down-regulated targeted DEGs in pJIA exerted function in interferon α/β, and those in AU are related to tyrosine kinases, Miro GTPases and RHOBTB3, RHOC GTPase, Rho GTPases, TGF-β, kidney development, I-κB kinase/NF-κB signaling, MAPK cascade, MHC class II antigen presentation, PI3K/AKT signaling, cell adhesion, and so on (**Supplementary Table 5**).

## 4 Discussion

The pathogenesis of JIA and AU has not been fully understood. One of the widely accepted hallmarks of the pathology of JIA is the expansion of inflamed synovial tissue or pannus, while synechia, edema, and angiogenesis are thought to be important in the pathology of AU (33). In this study, we found that most shared DEGs codes secreted proteins that exert their effects extracellularly which may have wide-reaching effects on multiple cells. It also fits the hypothesis that acquired and innate immunity disorders, which usually function by cell secretion, play important role in the common mechanism of pJIA and uveitis. We also found that not only IRGs correlated with both diseases but also up-regulated shared DEGs including TFs-targeted shared DEGs are associated with neutrophil degranulation.

Studies have shown that neutrophils could play important roles in autoimmune disease. It has been wildly understood that extracellular degranulation functions as a double-edged sword. The antimicrobials contained within granules can not only kill bacteria but also damage host tissue. Neutrophil granules contain 4 main types, namely secretory, tertiary (gelatinase), secondary (specific), and primary (azurophilic) granules. The primary granules contain neutrophil elastase, myeloperoxidase (MPO), azuracidin, and defensins. The secondary granules contain metalloproteinases (MMP), lactoferrin, cathelicidin, and lipocalin 2. The tertiary granules contain gelatinase, arginase 1, and lysozyme. Lastly, the secretory vesicles contain cytokines, albumin, and membrane receptors (Fc and complement receptors). Among them, primary granules that require the greatest stimulus for release contain the most pro-inflammatory and antimicrobial proteins, while secretory granules are released readily to replenish cell surface receptors. Additionally, most primary and secondary granule release is directed to the phagosome to minimize host tissue damage. Thus, the mechanisms that regulate neutrophil degranulation are important in maintaining proper immune homeostasis and avoiding tissue damage.

Initiation of neutrophil degranulation requires an adhesion-dependent signal that involves β2 integrins. Degranulation of azurophilic granules requires primed neutrophils that typically present enhanced generation of ROS by the NADPH oxidase. The NADPH oxidase complex whose morphology changed with the active status of neutrophil contains both RHO and Rac. We also found that up-regulated shared DEGs and TFs-targeted shared DEGs affect signalings of Rho GTPases, Miro GTPases, and RHOBTB3 (34, 35). Rho GTPases family including Rho, Rac, Cdc42, and RhoD/RhoF, are closely related to migration, adhesion, division, and polarity of cells (36), which are also important in neutrophil functioning. Fc receptors are an important component in the secretory vesicles and play part in initiating its activation. Functions disorders of FcγR and FcϵR may affect the degranulation of secretory vesicles, and impair the regulation of degranulation of the other 3 types of neutrophil granules. NF-κB is a pivotal mediator of inflammatory responses (37) whose activation cascade can be induced by lactoferrin, an important component in secondary granules (38). Other than that, a key component in primary granules, MPO, can induce neutrophil activation by MAPK and NF-κB activation. Thus, the dysregulation of NF-κB and MAPK pathways in pJIA and AU is also closely related to neutrophil functions, and neutrophil degranulation in particular.

It has been widely known that the imbalance of Th17, Th1, and Th2 cell and their related cytokines are important in the mechanism of autoimmune diseases. Studies have found that the main regulators of acquired and innate immunity are up-regulated in active JIA patients, including a group of interferon-induced genes. IFN-γ was significantly elevated in the serum of active patients, suggesting the imbalance of the Th1/Th2 immune response (39, 40). Previews studies have revealed that mechanisms of AU are closely related to enhanced activity of the IL-23/IL-17 pathway (41). Likewise, previous studies concerning noninfectious uveitis have found that various proinflammatory cytokines such as IL-1, IL-2, IL-6, IL-15, IFN-γ, TNF-α, IL-17, IL-23, IL-8, MCP-1, MIP-1β, IL-10, TGIF-β were elevated in serum, aqueous humor or vitreous (42). It is worth noting that IL-2, IL-6, IL-13, IL-18, IFN-γ, and TNF-α were significantly higher in the aqueous humor of children with JIA-associated autoimmune uveitis. Due to the higher incidence of AU onset, genetic studies mainly focused on RF-negative polyarthritis and oligoarthritis. Previous research concentrating on MHC found some shared alleles including also HLA, LXR, PD-1, PD-L1, IL-23R, TLR, PIP1, and PTPN (2). Moreover, IL-8, sICAM-1, and S100A12 have also been seen as potential biomarkers for JIA-associated autoimmune uveitis (43, 44). Our analyses of shared DEGs and IRGs indicated the disturbance of IL-2, IL-3, IL-4, IL-6, IL-10, and IL-13 are important in the pathogenesis of pJIA and AU too. These cytokines are all closely related to Th2 cells apart from IL-3, which stimulates hematopoiesis.

We also found potential roles in pJIA and AU of BCR, TCR, RUNX3, Class I MHC, RANKL/RANK signaling pathway, macrophage activation, calcium-mediated signaling, etc. Previous studies found shared HLA class I and class II loci alleles between AU and JIA indicating that both B cell and T cell functions are important in the common mechanism. Besides, neutrophils can activate naive CD8+ T lymphocytes *via* MHC class I antigen presentation as well as modulate B lymphocyte activation by secreting cytokines such as the B-cell-activating factor of the TNF family. RUNX3 is also involved in the development of CD8+ T lymphocytes. Furthermore, macrophages can acquire antigens *via* phagocytosis of neutrophils. RANKL/RANK is closely related to osteoclast by activation NF-κB pathway. Interestingly, several reports have described zoledronic acid that function by specifically suppressing NF−κB and JNK signaling could induce acute anterior uveitis (45). Previous study also found evidence showing that calcium-mediated signaling plays a key role when MPO stimulates neutrophils *via* the tyrosine kinase pathway.

Another interesting finding is that WGCNA of IRGs suggests that pJIA and AU are somehow related to kidney function like glomerular mesangial cell proliferation. Chronic kidney disease is a potential risk factor for uveitis based on a long-term population-based cohort study in TaiWan (46). Likewise, Gicchino et al. (47) found that about 8% of the children with JIA developed hypertension or chronic kidney disease. This is correlated with several previous reports suggesting that JIA patients could develop kidney damage due to renal amyloidosis or focal segmental glomerulosclerosis, etc (48, 49). These findings demonstrate the importance of periodic inspection of kidney function in those patients who committed pJIA and uveitis.

ARID1A, also known as BAF250A, is at the center of the TFs-shared DEGs network. It is a critical component of the mSWI/SNF complex and participates in numerous physiological processes including hematopoiesis, cell proliferation, migration, endothelial tube formation, angiogenesis, etc (50). Angiogenesis is considered to be an important early step in the pathogenesis of JIA (51), and is also involved in the mechanisms of uveitis (52). A recent study proved evidence that it’s also critical in JIA-associated autoimmune uveitis (53). It is worth noting that ARID1A was also found playing important role in T-cell development (54, 55), while its expression in B cells was found to regulate levels of IgE and IgG (56). Moreover, ARID1A has been found to induce antiviral IFN-I production in macrophages as well (57). It also exerts function in NF-κB and PI3K dependent manner. Those above-mentioned processes are all important in both pJIA and AU. Although today’s study about ARID1A mostly concerns malignant tumors, evidence of its role in autoimmune diseases like inflammatory bowel disease has been described recently (58).

We also found that both JIA and AU are related to hsa-miR-146, which was thought of as an NF-κB-dependent gene and an inhibitor that targets signaling proteins of innate immune responses. It could suppress pro-inflammatory cytokines in myeloid cells and T cells by regulating the expression of molecules in pathways including NF-κB. Interestingly, previous studies concerning pJIA and uveitis drew some controversial conclusions. Most studies found miR-146a was upregulated in JIA, but downregulated in uveitis.

MiR-146a was found to correlate with the disease activity of JIA like the JADAS score. Kamiya et al. also found a correlation between serum miR-146a and MMP-3 in pJIA patients (59). A possible explanation of the positive correlation between miR-146a and JIA might be that the increase of miR-146a aims to inhibit aberrant inflammation but could not block or reverse all the effects. Theoretically, with the up-regulation of miR-146a, its target genes should be downregulated. The up-regulated target DEGs of miR-146a in pJIA could the those whose expression failed to be repressed. And their functions are enriched in neutrophil degranulation, Fc, and interleukins, which also play roles in the pathogenesis of pJIA based on previous findings. Likewise, some previous research considered up-regulating of miR-146a as a secondary change to cope with immune disorders in JIA (60, 61).

Down-regulating of miR-146a was thought of as taking part in the mechanisms of AU. Targeted genes of miR-146a were thus up-regulated and enriched in previews mentioned underline pathogenesis of uveitis such as interferon α/β, interferon γ, Rho GTPases, Miro GTPases, and RHOBTB3, metalloprotease class of deubiquitinases, RUNX1. However, down-regulated targeted DEGs in AU are also related to some factors that are involved in the pathogenesis of AU such as NF-κB, TGF-β, RHOC GTPase, Rho GTPases, Miro GTPases, and RHOBTB3. It suggested that there should be other mechanisms in the pathogenesis that could compensate effects of miR-146a on those targeted genes. Functions of Rho GTPases are enriched in both up and down-regulated targeted DEGs. The reason might be their expression varies over time and space according to the findings of Watanabe et al. (62).

Our study demonstrated the flexibility and complexity of the immune system disorders involved in autoimmune diseases like JIA and AU. But the study has some limitations. Firstly, the sample size included in our study was quite small. Secondly, some possible confounders of the data were not included in the matrix files (like sex, age, age at onset, disease activity, batch, and so on) and thus the underline bias cannot be controlled. Thirdly, our results only indicate the importance of neutrophil degranulation in their common parthenogenesis, but the mechanism of neutrophil degranulation participating in the pathological process has not been thoroughly studied, which limits the theoretical basis of the application of its markers such as cathelicidin and MPO in the diagnostic process. Likewise, the effectiveness of the ARID1A and MiR-146a levels in the distinction of their associated comorbidities also needs to be verified.

## Conclusion

Taken together, neutrophil degranulation can be considered a shared pathogenic mechanism of pJIA and AU. Moreover, the roles of ARID1A and MiR-146a are worthy of further in-depth study and could be potential therapeutic targets. Other than that, the importance of periodic inspection of kidney function is also noteworthy.

## Data availability statement

Publicly available datasets were analyzed in this study. This data can be found here: https://www.ncbi.nlm.nih.gov/geo/query/acc.cgi?acc=GSE66936; https://www.ncbi.nlm.nih.gov/geo/query/acc.cgi?acc=GSE55319.

## Author contributions

JZ developed theoretical formalism, performed the analytic calculations. YW performed data curation, and visualization. JH supervised the project and is responsible for the overall content as guarantor. All authors contributed to the article and approved the submitted version.

## Supplementary material

Supplementary Table 3 Positive and negative correlation modules with pJIA or Uveitis.
